# Collider-based movement detection and control of wearable soft robots for visually augmenting dance performance

**DOI:** 10.3389/frobt.2024.1450177

**Published:** 2024-11-29

**Authors:** Patrick Twomey, Vaibhavsingh Varma, Leslie L. Bush, Mitja Trkov

**Affiliations:** ^1^ Department of Mechanical Engineering, Henry M. Rowan College of Engineering, Rowan University, Glassboro, NJ, United States; ^2^ Department of Theatre and Dance, College of Performing Arts, Rowan University, Glassboro, NJ, United States

**Keywords:** activity recognition, movement detection, colliders, wearable sensors, inertial measurement units (IMUs), soft robots, dance

## Abstract

The fusion of wearable soft robotic actuators and motion-tracking sensors can enhance dance performance, amplifying its visual language and communicative potential. However, the intricate and unpredictable nature of improvisational dance poses unique challenges for existing motion-tracking methods, underscoring the need for more adaptable solutions. Conventional methods such as optical tracking face limitations due to limb occlusion. The use of inertial measurement units (IMUs) can alleviate some of these challenges; however, their movement detection algorithms are complex and often based on fixed thresholds. Additionally, machine learning algorithms are unsuitable for detecting the arbitrary motion of improvisational dancers due to the non-repetitive and unique nature of their movements, resulting in limited available training data. To address these challenges, we introduce a collider-based movement detection algorithm. Colliders are modeled as virtual mass-spring-damper systems with its response related to dynamics of limb segments. Individual colliders are defined in planes corresponding to the limbs’ degrees of freedom. The system responses of these colliders relate to limb dynamics and can be used to quantify dynamic movements such as jab as demonstrated herein. One key advantage of collider dynamics is their ability to capture complex limb movements in their relative frame, as opposed to the global frame, thus avoiding drift issues common with IMUs. Additionally, we propose a simplified movement detection scheme based on individual dynamic system response variable, as opposed to fixed thresholds that consider multiple variables simultaneously (i.e., displacement, velocity, and acceleration). Our approach combines the collider-based algorithm with a hashing method to design a robust and high-speed detection algorithm for improvised dance motions. Experimental results demonstrate that our algorithm effectively detects improvisational dance movements, allowing control of wearable, origami-based soft actuators that can change size and lighting based on detected movements. This innovative method allows dancers to trigger events on stage, creating a unique organic aesthetics that seamlessly integrates technology with spontaneous movements. Our research highlights how this approach not only enriches dance performances by blending tradition and innovation but also enhances the expressive capabilities of dance, demonstrating the potential for technology to elevate and augment this art form.

## 1 Introduction

Dance is an art form that has been present in human society for thousands of years. From ancient tribal dances to contemporary styles, dance has always been a means of expression and communication. In recent years, the intersection of technology and the performing arts has given rise to innovative approaches aimed at pushing the boundaries of artistic expression. The amalgamation of soft robotics and wearable technologies has paved the way for a novel avenue of exploration, where human movements become a canvas for the synchronization of mechanical augmentation. The usage of technology is becoming an increasingly important aspect of modern dance with the abundance of insight it can provide to the dancers and the audience. For example, Moriaty and Sykes (2022) presented the use of haptic feedback systems for audiences to influence the direction of performance. Haptic pads have been used to allow the audience, especially the blind, to feel the performance through vibrations corresponding to the dancers’ movements (Lycouris et al., 2012). Danceroom Spectroscopy is a tool that uses quantum molecular dynamics algorithms and depth sensors to render human movements as energy landscapes in a simulated environment (Glowacki et al., 2012). Allen d’Ávila Silveira et al. (2022) have developed a soft robotic wearable device that guides lower limb movements to inspire and challenge dancers by constraining and enabling new performance possibilities. Translating dance movements into physical form of feedback or input requires complex algorithms with short computational times that may be addresses by developing novel techniques.

Motion tracking in the context of detecting movements during dance performances presents a multifaceted challenge that stems from the intricacies of human movement and the need for real-time responsiveness. Traditional external motion tracking systems that use cameras often struggle to accurately capture the dynamic and nuanced motions inherent in dance, where fluidity, rapid changes, and intricate gestures are integral components. To avoid issues of occlusion prevalent in optical motion capture, IMUs have been added to some external camera-tracked solutions (Matsuyama et al., 2021). Nam and Kim (2018) presented a dance training game that employs wearable devices and motion capture technology to analyze and replicate dance movements for health and fitness purposes. Recently, Ami-Williams et al. (2024) examined the challenges of capturing the movement of dancers wearing traditional African masquerade garments, and developed an efficient pipeline for digitizing and visualizing these performances using a combination of motion capture technologies. The general use of movement detection algorithms and human motion capture have been greatly influenced by other areas outside of dance. For example, the IMU motion capture is used in various applications including rehabilitation (Gu et al., 2023), animation (Roetenberg et al., 2009), and teleoperation of robots (Miller et al., 2004; Kobayashi et al., 2014; Zhu et al., 2022). IMU sensor-based tracking has been used in applications such as slip prevention through detection of gait perturbations (Trkov et al., 2019), or in kneel assist devices to reduce occupational hazards for construction workers on roofs (Chen et al., 2021b; Chen et al., 2021a). The challenge with tracking and responding to dance movements lies in developing a system that not only accounts for the diverse range of movements performed by dancers that include whole-body multi-degree-of-freedom motions but also operates seamlessly, in real-time, is compact, and can operate for the whole duration of performance.

Dance motions and movements of limbs can be detected based on various IMU sensors-based algorithms. One of the widely used methods to estimate upper limb use is by applying a specific threshold to the measured IMU acceleration (Subash et al., 2022). Meghji et al. (2019) developed an algorithm for tracking and quantifying change of direction in athletes using IMU sensor signals partly by using a piece-wise linear thresholding algorithm. Detecting turns among Parkinson disease patients has been performed using different thresholds for head, neck, and ankle orientations sensed by IMUs (Rehman et al., 2020). All of the above-mentioned algorithms use a threshold-based algorithms, which require simultaneously checking of multiple variables (i.e., linear and angular displacements, velocities, and accelerations) of multiple limb segments that is computationally intensive and requires setting multiple thresholds. A possible alternative approach is to capture motion dynamics through introducing virtual mass-spring-damper elements that capture the dynamic response and simplifies the detection algorithm as discussed in this study. This concept has been used in a few robotics applications in different scenarios. Virtual spring-mass-dampers were used as virtual restrictions between arm and end-effector of two robots to impart obstacle avoidance capability (Jin et al., 2004). Swarm cohesion was achieved between multiple non-holonomic mobile robots (Wiech et al., 2018) as well as spacecraft formation control was demonstrated (Chen et al., 2015) using virtual spring-mass-damper connections between individual entities. A concept of virtual spring forks has also been used to obtain realistic visual force feedback from objects manipulated in virtual environments (Koutek and Post, 2001). The virtual representation of mass-spring-damper elements may be used to reflect naturalistic reaction of attached objects.

Dance performances can visually augment human movements by using wearable technologies that can produce visual stimulus through lighting or changes in shape or size of wearable articulating objects. Soft actuators, composed of flexible, compliant materials can mimic such natural movements and have found applications in various fields, including robotics, medicine, and the arts. Unlike traditional rigid actuators, soft actuators provide safe and adaptive interaction with their environment, making them suitable for delicate tasks and wearable technologies (Shintake et al., 2016; Polygerinos et al., 2015), including safe, close interaction with the dancers. In the arts, soft actuators are employed to create dynamic and interactive installations that respond to audience presence or environmental changes. For example, collaboration between artists and engineers has led to utilizing soft robotics to develop kinetic sculptures that move and transform in fluid, organic ways, enhancing the sensory experience and engaging viewers more deeply (Jørgensen, 2019). These actuators enable artists to explore new forms of expression, pushing the boundaries of traditional media by incorporating movement and interactivity into their work. An example of soft actuators that can achieve such large deformations with pre-programmable deformations was enabled by origami-inspired design with soft (Li et al., 2017) or rigid panels (Robertson et al., 2021). This emerging approach has been used for the development of soft actuators used in this work to augment dancers’ main stage performance.

In this paper, we present a wearable system for visually augmenting dance performance through novel collider-based movement detection algorithm and control of wearable soft robots. The colliders function as a mass-spring-damper dynamic system which response is taken as the only parameter in the detection algorithm, thus reducing the number of variables to be tracked. Importantly, the colliders operate in a relative reference system thus present a particular advantage to overcome drift of inertial sensors to guarantee precise tracking over the long period of time. In addition, we implemented the hash chains to store the sequence of detected events that significantly reduce the time complexity of the detection algorithm compared to traditional logic tree conditions checking. The advantages of the algorithm were validate through several experiments demonstrating no affect of drift on detection, reduction in computational time, and accurate detection of dance movements.

The proposed system has been used for dance movement detection to control actuator contractions and lighting effects by changing the visible color and its intensity. This produced a unique organic aesthetic of the main-stage performance that was possible through the collaborative effort between artists and engineers. Overall, the main contributions of this paper are threefold: (i) we present a novel collider-based approach that uses a virtual mass-spring-damper system in a relative frame to capture dynamics of human limb using a single variable that is not affected by the drift of inertial sensors, (ii) we demonstrate that hash chain method significantly speeds up the detection time of complex movements compared to traditional logic tree condition checking, and (iii) we demonstrate the use of soft actuators and collider-based algorithm can be used for visually augmenting dance performances. In subsequent sections we elaborate on the technical materials and methods used in this work, the corresponding results, and a concluding discussion.

## 2 Materials and methods

### 2.1 Origami inspired actuator

**FIGURE 1:**
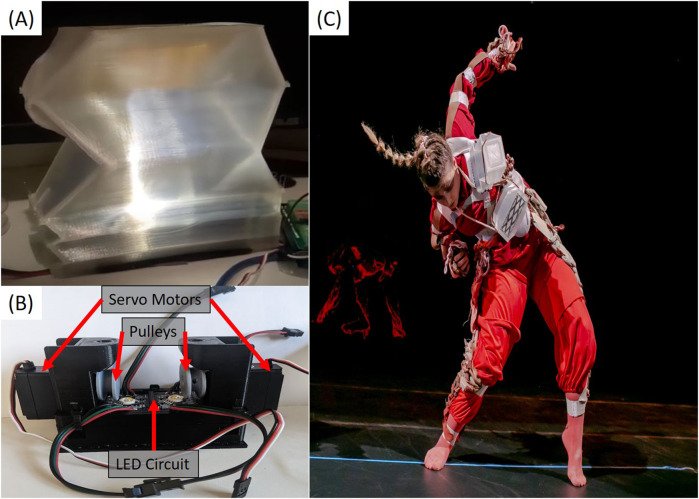
**(A)** Individal soft actuator along with **(B)** the internal pulley mechanism and LED circuit. **(C)** The soft actuators on a performer during the dance sequence.

The design of the soft actuators was iteratively developed through experiments. The actuators have to be large enough to be visible to the audience, while still allow dancers to perform improvisational movements. The initial simple inflatable pouch designs made of silicone rubber were omitted due to the required large amount of pressurized air for their operation making them unpractical. Thus origami-inspired structures were developed with electric motors to operate their contraction (see Figure 1). We fabricated the actuators out of translucent thermoplastic polyurethane (TPU) by 3D printing them on an Ender CR-10 FDM printer. An LED light was embedded inside as shown in Figure 1A, to provide additional visual effects and augment the dance performance. The main body of the actuator has a bellow-like structure that can fold upon itself. The actuator can be compressed by internal pulleys driven by two servo motors (see Figure 1B). Each actuator is independent and all the mechanical and electrical elements and circuits are contained within the TPU body. The actuators receive signals from the IMU trackers mounted on individual limb segments of a dancer and use them to control the desired actuator motions and LED color selection as guided by the colliders-based tracking algorithm described subsequently. Detected movements triggered simple on/off responses in the soft robots, which included both LEDs and electric motors. We did not incorporate proportional control, which could have allowed for more nuanced, continuous robot actions. Future work could explore more advanced control schemes involving multiple sequenced control gestures, enhancing the system’s responsiveness and adaptability.

### 2.2 Wireless IMU trackers

The IMU trackers are equipped with Bosch BNO055 and Bosch BMI270 sensors, designed for precise motion tracking. The Bosch BNO055 is a sophisticated 9-axis “absolute orientation sensor” that incorporates a 3-axis accelerometer, a 3-axis gyroscope, and a 3-axis magnetometer. It features a built-in micro-controller for sensor fusion, which processes raw data from these sensors to provide accurate orientation and motion tracking with minimal external processing. Conversely, the Bosch BMI270 is a low-power IMU that includes a 3-axis accelerometer and a 3-axis gyroscope, optimized for wearables and other battery-powered applications due to its energy-efficient design. The main reason for a built-in sensor redundancy is to increase robustness for magnetically unstable environments. In our implementation, we average signals from both sensors or the system can switch to the BMI270, if required due to presence of disturbance, to maintain reliable tracking. These sensors are paired with an ESP32-C3 micro-controller, which wirelessly transmits data to a Raspberry Pi running a local server for forward kinematics (FK) algorithms.

### 2.3 Forward kinematics

**FIGURE 2:**
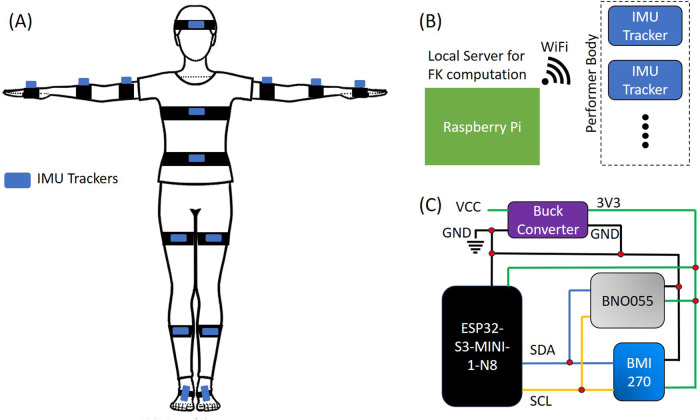
**(A)** Representation of a performer wearing the IMU trackers and standing in T-pose for calibration. **(B)** Schematic of the overall system. **(C)** Schematic electronic circuit of each IMU tracker.

Forward kinematics is used to convert the orientation data from the IMU trackers to limb and body positions. The full FK system consists of 15 tracking points, as illustrated in Figure 2. Trackers are attached to various segments of the body including - the head, the chest (upper torso), lower back (lower torso), biceps, forearms, hands, thighs, shins, and feet as shown in Figure 2A. The FK reference frame is positioned at the lower torso. For versatile use cases, the system allows enabling or disabling tracking for the upper and lower body if required.

During the initial system calibration, the user wearing the trackers assumes a T-pose (see Figure 2A). At this stage, an offset quaternion for each tracked limb is computed and saved. This calibration ensures accurate alignment of the virtual model with the user’s physical movements, facilitating precise motion tracking for applications ranging from movement analysis of dance performers to biomechanical human gait analysis. In operation, the Raspberry Pi server receives the quaternion values from all the IMU trackers. These quaternions are then converted to Euler angles - roll, pitch, and yaw, that define the orientation of each limb segment in space using Equation 1. Variables $q_{w},\;q_{x},\;q_{y},\;$ and $q_{z}$ are the quaternion components and ϕ,θ, and ψ are the roll, pitch, and yaw Euler angles, respectively. A unit vector in the direction defined by Euler angles and the limb lengths are then used to determine the position of the body in space.

$$\left[\begin{array}{c} \phi \\ \theta \\ \psi \end{array}\right]=\left[\begin{array}{c} atan2\left(2*\left(q_{w}*q_{x}+q_{y}*q_{z}\right),1-2*\left({q_{x}}^{2}+{q_{y}}^{2}\right)\right)\\ -\pi /2+2*atan2\left(\sqrt{1+2*\left(q_{w}*q_{y}-q_{x}*q_{z}\right)},\sqrt{1-2*\left(q_{w}*q_{y}-q_{x}*q_{z}\right)}\right)\\ atan2\left(2*\left(q_{w}*q_{z}+q_{x}*q_{y}\right),1-2*\left({q_{y}}^{2}+{q_{z}}^{2}\right)\right) \end{array}\right]$$ (1)

### 2.4 Colliders

**FIGURE 3:**
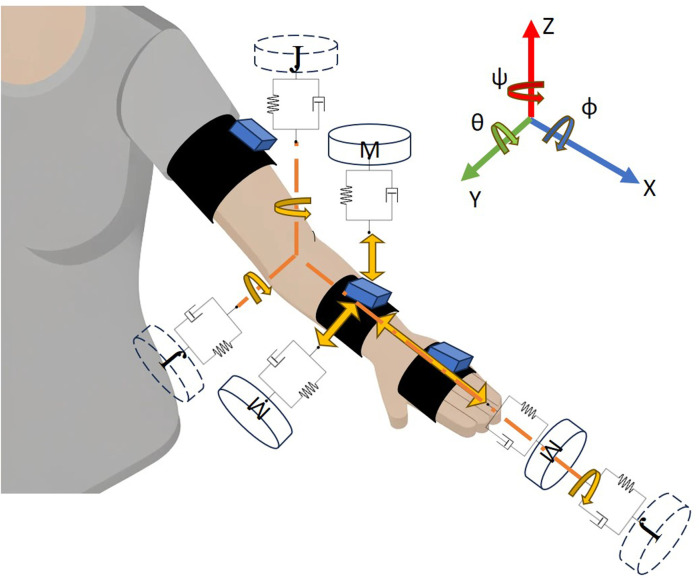
Representation of colliders related to the forearm IMU tracker and the corresponding elbow joint. Masses (M) shown with solid boundaries are for translation while the rotational inertias (J) with dashed lines are for rotational degrees of freedom.

Colliders provide a novel method for quantifying and detecting user movements based on measured limb kinematics, such as from wearable IMUs. The colliders function as virtual mass-spring-damper systems anchored to the current limb position of a subject (see Figure 3). They are defined in a relative coordinate system with respect to the individual limbs, which offers an important advantage of effectively mitigating sensor drift issues commonly present in inertial-based sensor measurements. Each collider is oriented in its respective axis representing linear or rotational degree-of-freedom. The limb motion is used as an input to the virtual mass-spring-damper systems and we observe its dynamic response. To detect a specific motion, we predefined a virtual space/bounds around the initial response signal and when collider’s dynamic motion intersects (i.e., collides) with that virtual bounds, it activates and enters a refractory period, during which it cannot be reactivated. The colliders’ responses inherently encompass dynamics associated with the position, velocity, and acceleration of the input (e.g., limb segment) that are instead combined in a single output response variable and thus reduce the number of tracking individual variables. Importantly, colliders maintain functionality regardless of potential sensor drift because their inputs are primarily based on relative velocity and acceleration. The following equations govern the mechanics behind movement detection using the colliders.

$$\left.M\ddot{q_{n}}=-C\left({\dot{q}}_{n-1}-{\tilde{\dot{q}}}_{n}\right)\right)-K\left(q_{n-1}-{\tilde{q}}_{n}\right)$$ (2)

In Equation 2, M represents the mass (or inertia) of the virtual collider, C is the damping, and K is the spring stiffness. $\ddot{q_{n}}$ , $\ddot{q_{n}}$ , and $q_{n}$ represent the collider’s response to the limb position ${\tilde{q}}_{n}$ , and the limb velocity ${\tilde{\dot{q}}}_{n}$ at any $n^{th}$ instant. The update for the collider state is obtained using simple integration Equation 3.

$$\begin{array}{cc} {\dot{q}}_{n} & ={\dot{q}}_{n-1}+{\ddot{q}}_{n}dt\\ q_{n} & =q_{n-1}+{\dot{q}}_{n}dt \end{array}$$ (3)

The collider-based detection is based on checking the relative position of the limb and the collider-response. A movement is detected once the collider state crosses a preset upper bound around the limb state and then the lower bound, in that sequence. This movement detection method is particularly ideal for use cases involving extended performances, such as dance routines, where traditional methods might require frequent re-calibration. Compared to conventional threshold-based approaches, colliders are adaptive, eliminating the need for setting static, predefined values for each individual. This adaptability significantly reduces setup time, as the process of determining actions associated with specific triggers only needs to be done once, enhancing efficiency and consistency in motion tracking. Collider performance is demonstrated and assessed in this study using a simple punch movements and through detecting dance movements. The detection of these events using colliders is compared to the detection using fixed thresholds. The method of colliders also offers an advantage in reducing the computational cost as large output sets bound by thresholds can be computationally expensive due to condition checking.

Colliders operate on a complexity scale of O(n) , where the computational load increases linearly with each new collider added, whereas fixed thresholds operate on O(n+1) , where the computational load increases with each new threshold added and an extra check for verifying if all conditions are being checked simultaneously. This makes colliders more efficient and scalable for complex motion-tracking systems.

### 2.5 Hash chains

**FIGURE 4:**
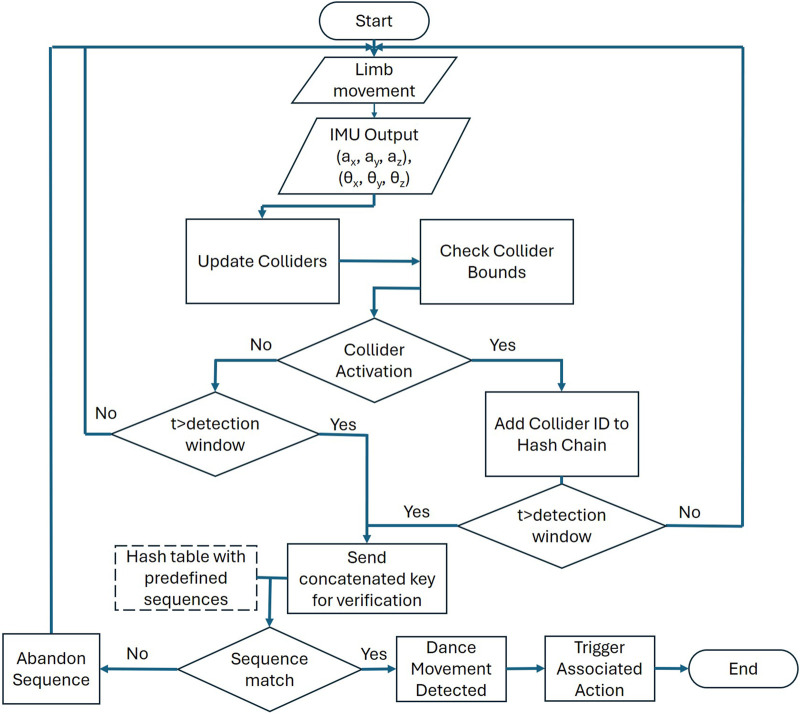
Schematics of a collider-based movement detection algorithm. The IMUs measure human kinematics and colliders are computed and checked for exceeding bounds. If a collider is activated within a set time detection window, t, its ID is added to a hash chain. The process repeats until no more activations occur within the time detection window, signaling the end of the chain. The completed chain is then sent to check the matching in the predefined hash table to identify the movement.

Detecting a sequence of motions was implemented using collider signals and hash chains method shown in Figure 4. Every time colliders are activated, they log a key into a queue and start a decay timer. If another collider is activated within this time frame, the new key is added to the queue, and this repeats until decay timer expires. When the decay timer expires without any new collider activations, all keys in the queue are concatenated and sent to a hash table for verification. If the concatenated key matches an entry in the predefined hash table, the associated action is triggered; if no match is found, the action chain is discarded. This process repeats for the duration of use.

The advantage of using a sequential hash is its average computational complexity of O(1) , meaning it performs in constant time regardless of the number of entries. In contrast, traditional logic trees have a complexity of O(n) , where the time required increases linearly with the number of actions. When comparing our combined system to traditional tracking setups, the latter typically have a complexity of $O\left(n^{2}+n\right)$ , making them significantly less efficient. Our proposed collider tracking with sequential hashes operates at O(n) , offering a more efficient and scale-able solution for complex motion tracking systems. We demonstrate performance comparison to validate the results in Section 3.3.

## 3 Results

### 3.1 Collider parameter sensitivity analysis

**FIGURE 5:**
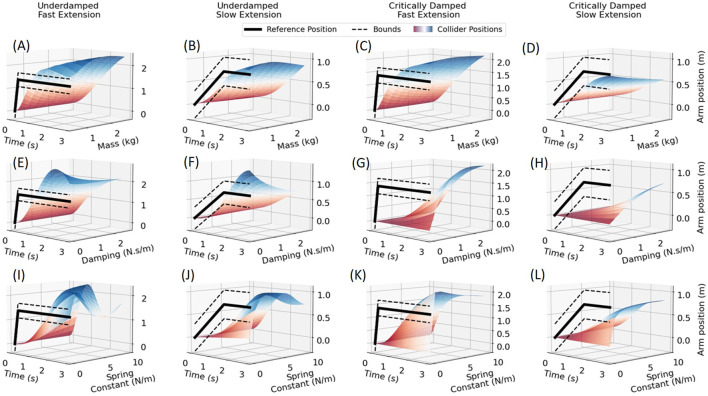
Collider responses for fast and slow arm punch movements for different combinations of collider’s mass, spring constant, and damping parameter. Effect of virtual mass variation is shown for **(A)** underdamped fast punch response, **(B)** underdamped slow punch response, **(C)** critically damped fast punch response, **(D)** critically damped slow punch response. Response of varying damping parameter is shown for **(E)** underdamped fast punch, **(F)** underdamped slow punch, **(G)** critically damped fast punch, **(H)** critically damped slow punch. Demonstration of spring constant variation on **(I)** underdamped fast punch response, **(J)** underdamped slow punch response, **(K)** critically damped fast punch response, **(L)** critically damped slow punch response.

Colliders are considered virtual mass-spring-damper systems. The selection of parameters associated with this system will affect how actions are detected in this context. Therefore, we performed a parameter sensitivity analysis to demonstrate the changes in system response and use them as a guidance for selection of parameters. We selected a simple arm extension action as a reference to evaluate the response of the colliders by varying virtual mass, spring constant, and damping parameters. Figure 5 shows various cases that were evaluated to finalize a set of parameters that would work well for the purpose of the pertinent dance performance. Underdamped (Figures 5A, B, E, F, I, J) and critically damped (Figures 5C, D, G, H, K, L) conditions are shown for fast and slow extension movements, simulating a straight arm punch, wherein the hand position varies from 0 (initial position) to full arm extension 0.77 m and held for 3 s. Mass and damping were both varied from 0 to 3 and spring constant from 0 to 10 in their corresponding units. When varying one quantity the other two were fixed at 1 in underdamped case and appropriately calculated for critical damping case using the equation: $C=2\sqrt{MK}$ , where C is the damping, K is the spring constant, and M is the mass.

It is clear from Figure 5 that the critically damped response is slow and does not reach the reference arm position. Therefore, this condition is not ideal for movement detection of real-time performances. Underdamped condition with mass 1 kg, damping 1.5 N s/m, and spring constant 9 N/m were selected. Underdamped system response is much faster, crosses the reference signal, and is used for detecting crossing of both upper and lower bounds around the reference signal as well as the sequence in which they are crossed. These sequences make for simpler conditions to be checked for detection. For instance, if the sequence of bounds being crossed is lower-upper-lower it is easy to conclude that the arm was extended and withdrawn. Similar simple sequences can be created for all types of movements based on the reference and bounds even for continuous cases.

### 3.2 Colliders in long performances not affected by drift

**FIGURE 6:**
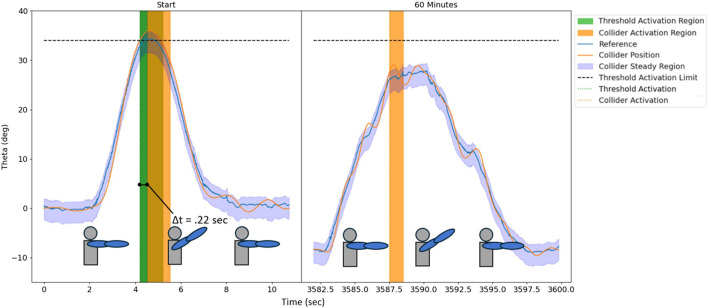
Impact of IMU sensor drift on motion detection and demonstration of successful functioning of colliders in a drift-affected system where traditional thresholds fail to detect events. At the start of a test, fixed threshold and collider-based algorithm both correctly detect the motion (i.e., shoulder extension); left plot. After 60 min of data collection and presence of drift in a reference signal, fixed threshold does not detect the event, while collider successfully detects the motion; right plot.

Drift is one of the leading reasons for errors when using IMU sensors over a long period of time that affect sensor signals. Contrary, colliders are bound to the reference and the colliders’ origin moves along with the sensor signal. In cases like dance movement detection, where the exact joint angle measurement are not of primary goal and detecting dance movements is the primary focus, colliders can help offset the effect of drift. Fixed thresholds may either miss or incorrectly detect the movement if a sensor signal has drifted over time. We evaluated the effect of drift by detecting a similar arm-raise movement initially when the sensors were turned ON and then about 60 min later after allowing the sensors to drift. The results of this evaluation are shown in Figure 6. The left side of the figure shows the first movement at the start during the first 10 s. It can be seen that the signal crosses fixed threshold for this movement and both fixed threshold and collider method detect the movement. However, on the right side of the figure when the action is repeated after 60 min, the sensor has drifted (8 deg), and the fixed threshold is never crossed meaning movement was not detected. Contrary, since the collider’s origin moves with the reference signal, the collider is still able to detect the movement. The signal used here is the shoulder extension angle involved in raising the arm. The collider detects the movement at about 0.22 s later than the fixed threshold at the start because the collider has to make a fixed number of mandatory checks of the collider state. However, with colliders and hash chain implementation, the execution time remains constant even if the number of movements to be detected (conditionals) increases. The subsequent section discusses the advantage of hash chains as conditionals increase.

### 3.3 Performance comparison of hash chain with traditional logic tree checking

**FIGURE 7:**
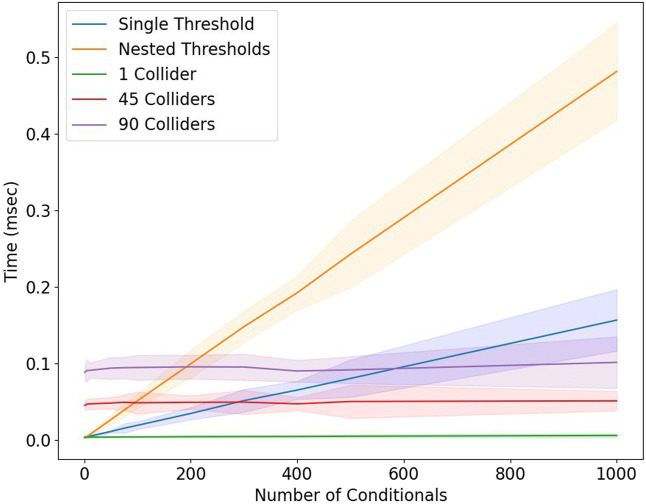
Comparison between the performance of fixed thresholds with decision statements and colliders with hash chains for detecting movements represented by the mean time taken for execution and the corresponding standard deviation as the number of conditionals is increased.

Hash chains used with colliders provide a way to maintain the time complexity of the movement detection algorithm constant even when the total number of actions/conditionals is increased. Traditional if statements with single threshold and nested threshold checks when evaluated for increasing the number of conditionals clearly show a linearly increasing time requirement as seen in Figure 7. The single threshold checks involve checking only one type of movement at a time. Whereas the nested threshold checks evaluate compound actions comprised of multiple movements happening in a particular sequence. In the current scenario, three types of threshold checks were evaluated with each check consisting of conditionals in a range of 0–1,000. For nested thresholds case, the conditionals were nested inside each threshold check. In Figure 7, the standard deviation also increases due to the fact that the time of execution varies between the fastest and slowest depending on the number of conditionals being checked. It is also clear that the collider-based method with hash chains executes with constant time even with an increase in the number of colliders and the number of conditionals in the hash chain. The maximum number of colliders possible for our wearable system is 90, with a maximum of 15 trackers per subject and with 6 colliders for each limb tracker. The collider method has a constant performance even with zero conditionals because there are a set number of mandatory checks to be conducted based on the number of colliders. While colliders outperform the fixed threshold checking, even the worst-case collider and hash chain method performance is seen to be better than that of the threshold method as the number of conditionals increases beyond 200. This makes colliders and hash chains better for real-world performance situations where the types of distinct movements can be very high.

### 3.4 Detection of dance movements using colliders

#### 3.4.1 Arm punch detection

**FIGURE 8:**
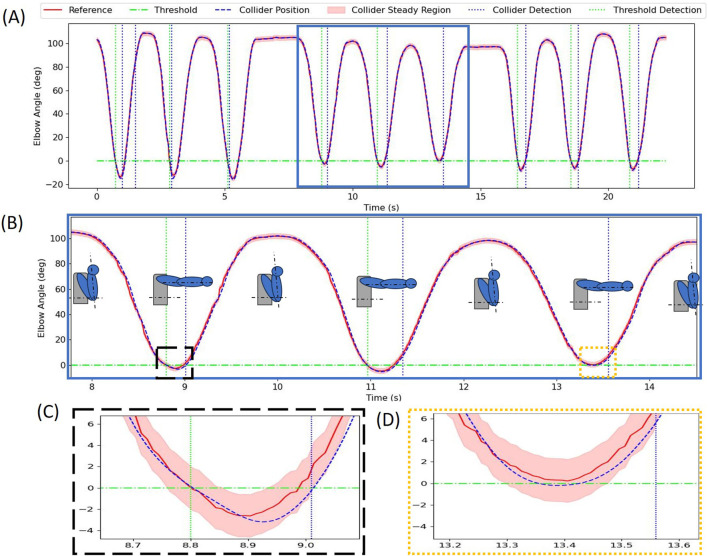
**(A)** Detection of nine consecutive straight arm punch movements using fixed threshold and collider methods. **(B)** Detail showing detection for three consecutive punches with representative limb positions. **(C)** Detail showing detection using both threshold and collider-based methods. **(D)** Detail showing instance when fixed threshold method does not detect one of the punches (fixed threshold is set at 0 deg), while collider method successfully detects the movement.

Detection of specific movements was first demonstrated on a simplified example of a straight-arm punch. The movement was detected based on the extension of the elbow. Nine repeated movements were completed and recorded. Figure 8 shows the detection of the punch using a rotational collider at the elbow joint and fixed threshold. The punch is detected when the arm is fully extended and the elbow is almost parallel to the horizontal reference as shown by the schematic poses in Figure 8B. Both the collider and fixed threshold algorithms detect the first two movements; however, the last punch does not cross the threshold and is only detected by the collider. The limitation of using a fixed threshold is seen in Figure 8D where the signal misses the threshold by a very small margin and is not detected. Figure 8C shows the detection delay of the collider as compared to the fixed threshold method. The delay is 0.2 s for the first and 0.37 s for the second punch. The collider always detects the punch by checking the following sequence wherein the collider signal first crosses the upper bound (see Figure 8B), then the lower bound of the collider steady region (see Figures 8C, D). The underdamped nature of the collider allows for this behavior of the collider signal and it can be extended to all kinds of movements by selecting any appropriate limb angle or translation obtained from the kinematics computation.

#### 3.4.2 Dance movement detection

**FIGURE 9:**
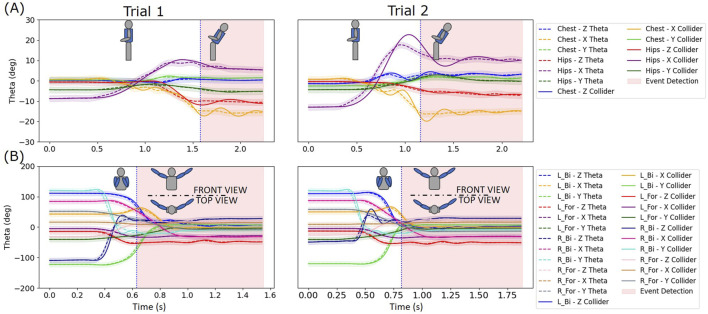
Detection of actual actions performed by two separate subjects. **(A)** Detection of back bending backwards using 6 colliders (i.e., Chest XYZ and Hip XYZ). **(B)** Detection of double full arm extension out to the side from arms tucked in position using 12 Colliders (i.e., Left Bicep XYZ, Left Forearm XYZ, Right Bicep XYZ, Right Forearm XYZ).

Two different dance poses were mimicked by two different subjects simulating movements performed by dancers, bending backward and full arm extension out to the sides from a tucked-in position. The back bending portion was detected using six rotational colliders with three colliders (one in each rotational axis) respectively placed on the hip/lower back and upper back segments (i.e., Chest XYZ and Hip XYZ). The arm extension was detected using twelve rotational colliders such that one collider was placed on each axis (i.e., X-, Y-, and *Z*-axis) of the left bicep, left forearm, right bicep, and right forearm. Figure 9 shows the detection of dance pose events using these colliders. The detection event is registered as the performer comes to the limits of their pose and begins to hold the pose.

As seen in Figure 9A the peaks of the angles about *X*-axis are good indicators of the back bending movement and the collider detects this just after the peak when the collider signal has registered the required sequence of keys similar to those described in the previous section describing the simple arm punch movement. Similarly, in Figure 9B the collider detects the movement after all the keys for all the involved angles are satisfied in the desired sequence. The checking of all involved collider signals for keys proves to be a simple and effective way to detect compound movements involving multiple limbs. Importantly, successful detection results also shows that the same set of colliders can be used for different performers without the need for making a new set of thresholds, which significantly simplifies the implementation and emphasizes practicality of this approach.

## 4 Discussion

The motivation behind this work was the requirement for movement detection for a dance performance. Soft actuators were developed to depict reactive naturalistic motions based on the movements of the performer. We acknowledge that our motions were not performed by the dancers and were simulated to replicate the motion observed. Regardless, the results demonstrate the successful motion detection on data from two subjects that validates the proposed algorithm.

The results present the use and advantage of colliders over fixed thresholds. Initially, it is necessary to select a set of parameters for the colliders to suit the type of application. Therefore, an analysis of collider behavior with varying virtual mass, spring constant, and damping values is presented and an underdamped system with 1 kg mass, 9 N/m spring, and 1.5 N s/m damping was selected as it gives a fast response. The fast response as well as overshoot was desired for movement detection in the current application of dance movements. An expected behavior of a second-order underdamped system with an overshoot is purposely utilized to set the expected sequence of detecting when the system response crosses collider’s steady region. Importantly, the collider’s response always follows the reference movement. This characteristic was beneficial when considering the case where sensors may drift in magnitude over time. Even with the drift of magnitude of a movement, the movement pattern and associated dynamics are preserved, which preserves the functionality of a collider and performs check of its position signal with respect to its upper and lower bounds of collider’s steady region to successfully detect the movement. Whereas, a fixed threshold which is set at the start may not be able to detect the movement if the sensors drift during a performance. Lakshmiprabha et al. (2015) proposed a method for drift reduction of IMUs using sensor fusion with vision data with an extended Kalman filter, at regular intervals; however, they report that the method may not be robust enough to account for different types of drifts for different IMU sensors. Another method, which uses local IMU accelerations for dead-reckoning drift reduction to estimate kinematic chain (like the human body) positions in space-like environments, is limited is limited and cannot be used for our application due to by presence of gravity and considering slow movements (Stretton and Koulieris, 2024). The method introduced in this work also presents limitations in the fact that different environments will require different collider parameters for accurate results.

Colliders are also implemented along with hash chains to store the movement sequences and to register multiple movements that are performed in series. This implementation has a constant time of execution even if the number of movements/conditionals to be detected increases. With fixed thresholds, checking for each condition and a sequence of conditions takes increasingly more time. This advantage of the colliders and hash chain method is particularly clear when there are more than 200 unique movements/conditionals. In the detection of movements, we implemented a simple straight-arm punch movement which showed how the fixed threshold is not able to detect the movement as the reference signal does not cross the threshold line. This specific example shows another important advantage of the collider signal which continuously follows the reference signal and will always detect the movement even if the magnitudes change. In other words, this means that it is not necessary for each punch to be executed in the exactly same manner with the total arm extension, as the colliders can capture the essence of the movement even for shorter punches, as demonstrated in Figure 8.

Increasing the number of conditionals for situations with large number of involved variables and conditions, increases complexity of movement detection and computational time (see Figure 7). The hash chain algorithm is perfectly suitable for such applications as the computational time remains constant. Event keys representing sequence of collider detection can be defined for various movements and checked simultaneously in specific instance of time. In our specific demonstration, he backwards back bending and double-arm extension movements shown in Figure 9B were presented as an example of detecting compound movements using multiple colliders simultaneously by checking simple keys. These keys can be further expanded in the future for detecting multiple events of movements and used in dance performances or general event detection.

We acknowledge that the presented method has also limitations. For example, collider detection is slightly delayed as compared to the fixed threshold detection (see Figures 8, 6); however, this was not considered as significant in the present context and our specific application. Nevertheless, the colliders can be tuned to detect earlier by making the virtual mass-spring-damper system more underdamped (i.e., decreasing damping ratio $\zeta =\frac{C}{2\sqrt{MK}}\ll 1$ ), which will increase the speed of colliders’ response as demonstrated in Figure 5. Another potential limitation is that the inertia of the actuators with respect to the limb motion may induce noise in the sensor reading and could affect the algorithm accuracy. As per feedback from the dancers, one of the constraints that the soft structures imposed on dancers was that they limited some specific dance movements that could not be performed due to the placement of the actuators at specific body locations. For example, rolling over on the side was not possible if the actuator was placed on the arm. In addition, wired IMUs were replaced with wireless modules at the initial stages of the study, to facilitate better dance movements.

In summary, this paper presents a novel virtual collider-based movement detection approach. The colliders, modeled as virtual mass-spring-damper elements, were shown to have the capability of detecting naturalistic movements in real time for artistic performances or other movements. The audience was able to appreciate the use of this technology for an immersive experience during a recent dance performance. In the context of our application, the gesture-based control system creates a versatile toolbox of effects, each tied to specific gestures that dancers can naturally integrate into their movements. This setup ensures responsiveness, allowing dancers to trigger effects, and create visual augmentation using technology during a dance performance, reliably without needing to worry about exact positioning or constant re-calibration during their performance. Beyond dance, this system could have applications in the fields such as healthcare, enabling gesture-controlled assistive devices, or gaming, where it could offer more immersive virtual reality experiences. In addition, the proposed system can be used in the industrial settings, where gesture control could streamline machine operation, providing an intuitive and efficient interface. Its flexibility makes it a powerful tool for enhancing user interaction across a wide range of fields.

## Data Availability

The raw data supporting the conclusions of this article will be made available by the authors, without undue reservation.
